# Genome-wide identification of MBD gene family members in *Eleutherococcus senticosus* with their expression motifs under drought stress and DNA demethylation

**DOI:** 10.1186/s12864-023-09191-x

**Published:** 2023-02-22

**Authors:** Shuo Wang, Jing Dong, Xue-Lei Zhao, Xin Song, Yue-Hong Long, Zhao-Bin Xing

**Affiliations:** College of Life Sciences, North China University of Science and Technology, Tangshan, China

**Keywords:** Methyl-binding domain protein, *Eleutherococcus senticosus*, Cis-acting element, Motif

## Abstract

**Background:**

Methyl-binding domain (MBD) is a class of methyl-CpG-binding domain proteins that affects the regulation of gene expression through epigenetic modifications. *MBD* genes are not only inseparable from DNA methylation but have also been identified and validated in various plants. Although *MBD* is involved in a group of physiological processes and stress regulation in these plants, *MBD* genes in *Eleutherococcus senticosus* remain largely unknown.

**Results:**

Twenty *EsMBD* genes were identified in *E. senticosus*. Among the 24 chromosomes of *E. senticosus*, *EsMBD* genes were unevenly distributed on 12 chromosomes, and only one tandem repeat gene existed. Collinearity analysis showed that the fragment duplication was the main motif for *EsMBD* gene expansion. As the species of Araliaceae evolved, *MBD* genes also evolved and gradually exhibited different functional differentiation. Furthermore, cis-acting element analysis showed that there were numerous cis-acting elements in the *EsMBD* promoter region, among which light response elements and anaerobic induction elements were dominant. The expression motif analysis revealed that 60% of the *EsMBDs* were up-regulated in the 30% water content group.

**Conclusions:**

By comparing the transcriptome data of different saponin contents of *E. senticosus* and integrating them with the outcomes of molecular docking analysis, we hypothesized that *EsMBD2* and *EsMBD5* jointly affect the secondary metabolic processes of *E. senticosus* saponins by binding to methylated CpG under conditions of drought stress. The results of this study laid the foundation for subsequent research on the *E. senticosus* and *MBD* genes.

**Supplementary Information:**

The online version contains supplementary material available at 10.1186/s12864-023-09191-x.

## Background

*Eleutherococcus senticosus* (Rupr. et Maxim) Maxim, also known as Siberian ginseng, is a valuable medicinal plant [1]. The extracts of its roots and bark have various physiological effects, such as modulation of the immune response [2]. Among the various bioactive constituents of *E. senticosus*, triterpenoid saponins, flavonoids, coumarins, and lignans play important roles in regulating these physiological activities [3]. The farnesyl pyrophosphate synthase [4] (FPS), squalene synthase (SS), squalene epoxidase [5, 6] (SE), and mevalonate pyrophosphate decarboxylase [7] (MDD) of *E. senticosus* are key enzymes that catalyze the biosynthesis of triterpenoid saponins. In previous studies, it was found that DNA methylation in the promoter regions of *FPS*, *SS*, *SE*, and *MDD* [7, 8] genes in *E. senticosus* inhibited the synthesis of the mevalonate pathway, thus reducing the accumulation of saponins.

Drought stress is usually considered a negative factor in plants and an important cause of yield loss. However, studies have shown that plants exposed to drought stress accumulate higher concentrations of secondary metabolites [9]. For example, drought stress dramatically decreases spica biomass production but increases rosmarinic acid (RA), ursolic acid (UA), and oleanolic acid (OA) contents in *Prunella vulgaris* L. [10]. In *Salvia miltiorrhiza* Bunge, water stress significantly increased the salvianolic acid B yield and decreased that of tanshinone IIA [11]. However, there are no reports on drought stress in the Araliaceae family. Drought stress is a type of adversity stress, in which plants respond to the external environment through various mechanisms. DNA methylation is one of the earliest and most studied mechanisms that regulate genome function and induce plant resistance and abiotic stress adaptation [12]. This provided a new idea for our research. Because the secondary metabolism of medicinal plants is affected by drought stress, they are inevitably subjected to epigenetic modifications. However, it is unclear how plants affect metabolite accumulation through epigenetic modifications under drought conditions.

DNA methylation is an epigenetic modification that plays an important role in regulating gene expression. Methyl-binding domain (MBD) protein is a class of methyl CpG-binding domain protein that selectively bind to fully methylated CpG dinucleotides and participate not only in the recruitment of nucleosome remodeling factors and histone deacetylases, but also in the formation of transcriptional repressor complexes that regulate gene expression through epigenetic modifications [13]. In humans, abnormalities in the *MBD* genes are strongly associated with the development of cancer and psychiatric disorders [14]. For example, overexpression of *MBD1* in the human body is usually associated with carcinogenesis, leading to the silencing of tumor suppressor genes through the formation of the abovementioned complexes [15]. In plants, *MBD* genes also play important functions: in *Arabidopsis thaliana* (L.) Heynh. *AtMBD4* mediates phosphates uptake [16]. Similarly, *AtMBD6* is involved in RNA-mediated gene silencing [17], whereas *AtMBD9* is involved in regulating flowering time and shoot branching in *A. thaliana*, with mutant plants exhibiting an abnormal phenotype of early flowering and branching [18]. These studies on *MBD* have demonstrated their importance. Therefore, a holistic analysis of *EsMBD* in *E. senticosus* could provide a new research perspective for exploring the mechanism of DNA methylation of secondary metabolism-related genes.

## Results

### Identification and analysis of the physicochemical properties of the *EsMBD* gene family

Given the Hidden Markov Model of the *MBD* gene family (PF01429), 20 *EsMBD* genes were screened out from the whole-genome sequencing data of *E. senticosus*, and sequentially named *EsMBD1-EsMBD20* according to their individual positions on the chromosomes (Table 1). Based on the Hidden Markov Model of the *MBD* gene family, 10 *AeMBD* genes were screened from the whole genome sequencing data of *Aralia elata* (Miq.). Seem and consecutively named *AeMBD1-AeMBD10* according to their separate positions on the chromosomes. The results showed that the quantity of amino acids in the *EsMBD* gene family of proteins was in the range between 166 and 2,015 aa, and the molecular weight ranged from 18,865.60 to 226,144.33 Da, whereas the isoelectric points were below 7.00 except for *Es*MBD7 (9.27), *Es*MBD11 (9.39), *Es*MBD14 (8.68), *Es*MBD15 (7.08), and *Es*MBD17 (8.27), indicating that most *Es*MBD proteins were acidic. Moreover, all *Es*MBD proteins exhibited hydrophobicity of less than 0 and an instability index greater than 40, indicating that all *Es*MBD proteins were unstable and hydrophilic.

**Table 1:** Physicochemical properties of *Es*MBD gene family proteins

Gene Name	Gene ID	Quantity of amino acids/aa	Molecular weight/Da	Isoelectric point	Hydrophobicity	Instability index
*Es*MBD1	Ese01G001833	578	63613.66	5.25	-0.784	44.26
*Es*MBD2	Ese01G003936	339	37649.14	4.77	-1.297	54.04
*Es*MBD3	Ese03G001766	372	41867.69	5.22	-0.749	53.35
*Es*MBD4	Ese03G000746	1248	138135.81	5.10	-0.651	45.63
*Es*MBD5	Ese03G002722	338	37539.06	4.75	-1.256	43.79
*Es*MBD6	Ese03G003163	578	63631.68	5.29	-0.810	45.48
*Es*MBD7	Ese04G003192	316	35944.76	9.27	-0.708	60.01
*Es*MBD8	Ese08G003485	263	28964.99	5.15	-1.351	57.78
*Es*MBD9	Ese08G002921	368	41838.60	5.51	-0.785	59.19
*Es*MBD10	Ese09G002039	2015	226144.33	5.40	-0.459	47.53
*Es*MBD11	Ese10G001996	209	23363.52	9.39	-0.515	58.57
*Es*MBD12	Ese11G001419	228	25831.55	6.05	-1.177	52.80
*Es*MBD13	Ese12G002522	382	43366.30	5.11	-0.731	61.24
*Es*MBD14	Ese12G002001	937	103828.32	8.68	-0.810	51.91
*Es*MBD15	Ese13G002475	967	108033.86	7.08	-0.859	50.66
*Es*MBD16	Ese14G002218	332	36570.91	4.78	-1.423	52.80
*Es*MBD17	Ese17G001961	166	18865.60	8.27	-0.575	48.00
*Es*MBD18	Ese17G000257	232	26081.80	6.22	-1.219	54.92
*Es*MBD19	Ese17G000341	384	41510.17	4.48	-1.143	54.80
*Es*MBD20	Ese18G001412	345	38815.07	4.44	-0.494	40.79

### Chromosomal localization and collinearity analyses of *EsMBD* genes

**Fig. 1:**
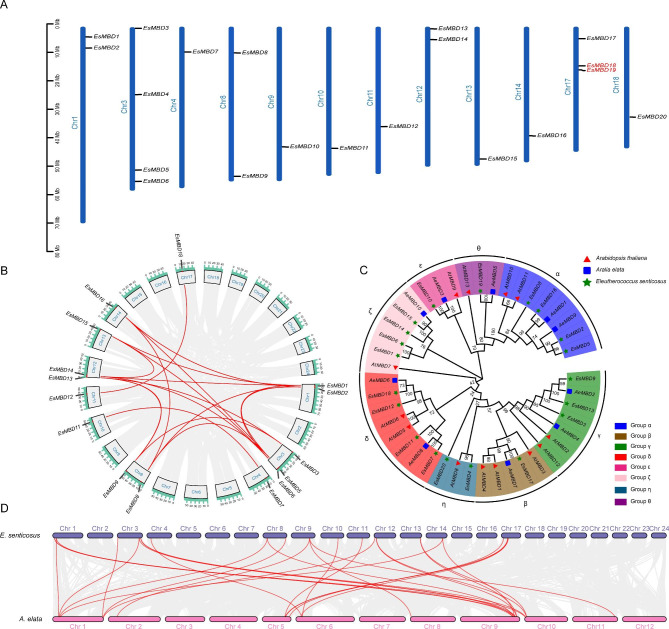
Chromosomal localization, evolutionary analysis, and collinearity analysis of *EsMBD* genes in *E. senticosus*. A: Chromosomal localization of *EsMBD* genes in *E. senticosus*. Red markers indicate tandem repeats; B: Collinearity analysis of *EsMBD* genes of *E. senticosus*; C: Phylogenetic analysis of *EsMBD* genes of *E. senticosus;* and D: Collinearity analysis of interspecies *MBD* between *E. senticosus* and *A. elata*. Red lines indicate the *MBD* genes

The chromosomal localization analysis of *EsMBD* genes (Fig. 1A) showed that they were unevenly distributed in 12 of the 24 *E. senticosus* chromosomes. Chromosome No. 3 contained four *EsMBD* genes, chromosome 17 contained three *EsMBD* genes; chromosomes No.1, 8, and 12 each contained two *EsMBD* genes; and chromosome No.4, 9, 10, 11, 13, 14, and 18 each contained one *EsMBD* gene. The distribution of *EsMBD* and tandem repeats on different chromosomes of *E. senticosus* are shown in Fig. 1A, in which only one pair, *EsMBD18* and *EsMBD19*, were tandem repeat genes.

The results of the collinearity analysis of *EsMBD* genes in *E. senticosus* (Fig. 1B) showed 22,568 genes in the genome, accounting for 62.05% of all genes, and collinearity was observed among the 17 *EsMBD* genes in the entire family. As shown in Fig. 1B, in the *E. senticosus* genome, these genes were of collinearity: *EsMBD1/EsMBD6*, *EsMBD1/EsMBD14*, *EsMBD1/EsMBD15*, *EsMBD2/EsMBD5*, *EsMBD2/EsMBD8*, *EsMBD2/EsMBD16*, *EsMBD3/EsMBD9*, *EsMBD3/EsMBD13*, *EsMBD5/EsMBD8*, *EsMBD5/EsMBD16*, *EsMBD6/EsMBD14*, *EsMBD6/EsMBD15*, *EsMBD7/EsMBD11*, *EsMBD8/EsMBD16*, *EsMBD9/EsMBD13*, *EsMBD12/EsMBD18*, and *EsMBD14/EsMBD15*, which presented many fragmental repeats among the *EsMBD* gene family.

The results of the collinearity analysis between the *EsMBD* genes of *E. senticosus* and the *AeMBD* genes of *A. elata* (Fig. 1D) showed significant collinearity between *E. senticosus* and *A. elata*, between *EsMBD* and *AeMBD* genes, and between the same *EsMBD* and different *AeMBDs*. This indicated that *MBD* genes evolved with the evolution of species in Araliaceae.

### Phylogenetic analysis of MBD

The 20 *Es*MBD protein sequences identified from the screening and 10 *Ae*MBD sequences from *A. elata* were used to construct a phylogenetic evolutionary tree, in addition to the 13 *At*MBD protein sequences with well-defined functions in *A. thaliana*. The results (Fig. 1C) showed that the evolutionary tree was divided into eight subfamilies, α, β, γ, δ, ε, ζ, η, and θ, based on the affinities between the different members of the MBD gene families of *E. senticosus*, *A. elata*, and *A. thaliana*. Except for the η subfamily, which contained only *EsMBD4, EsMBD20 * and *AtMBD8*, the *MBD* genes of *E. senticosus* and *A. elata* were both localized in the remaining subfamilies. The α subfamily contained 4 genes, *EsMBD2*, *EsMBD5*, *EsMBD8*, and *EsMBD16;* the β subfamily contained 1 *EsMBD17* gene; the δ subfamily contained *EsMBD7*, *EsMBD11*, *EsMBD12*, and *EsMBD18;* and the γ subfamily contained *EsMBD3*, *EsMBD9*, and *EsMBD13*. The δ subfamily contained *EsMBD7*, *EsMBD11*, *EsMBD12*, and *EsMBD18;* the ε subfamily contained *EsMBD10;* and the ζ subfamily contained *EsMBD1*, *EsMBD6*, *EsMBD14*, and *EsMBD15*. The η subfamily contained *EsMBD4* and *EsMBD20*, whereas the θ subfamily contained only *EsMBD19*.

### Structure of *EsMBD* genes with analyses of conserved structural domains and motifs of the protein

**Fig. 2:**
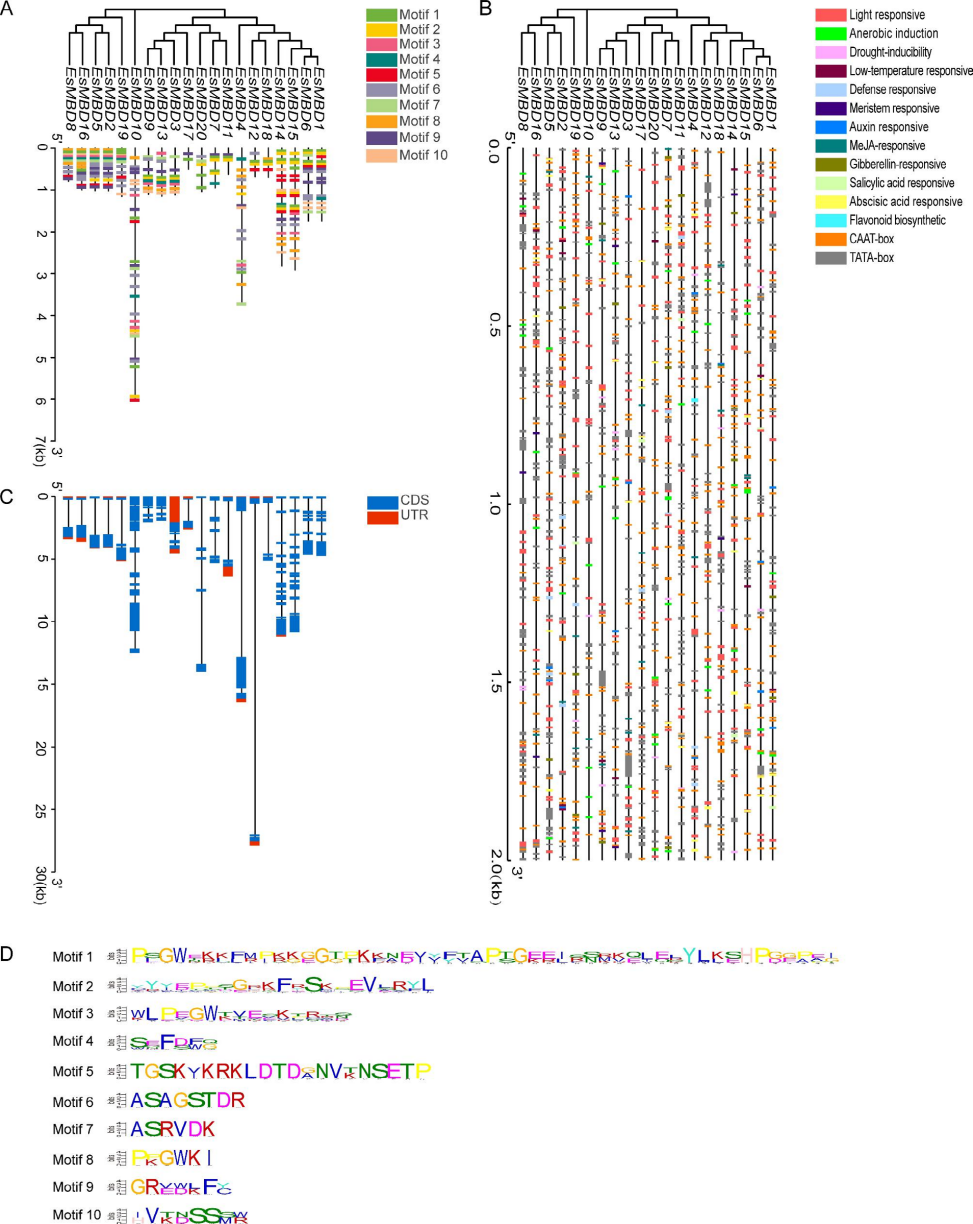
Gene structure, promoter analysis, and conserved motifs of *EsMBD* in *E. senticosus*. A: Motif analysis of *EsMBD* genes in *E. senticosus*; CDS: DNA sequence corresponding to the protein sequence one by one; UTR: non-coding segment at both ends of the mRNA molecule B: Promoter analysis of *EsMBD* in *E. senticosus*; C: Exon-intron structure analysis of *EsMBD* genes in *E. senticosus*; D: Conserved motifs of *EsMBD* genes in *E. senticosus*; Motif: conserved sequences with biological functions

The structure of *EsMBD* genes in *E. senticosus* is shown in Fig. 2A C. Of the *EsMBD* gene sequences, 70% were within 2,000 bp in length, only one was up to 6,000 bp in length, and only 20% were approximately 1,000 bp in length. Ten genes in the *EsMBD* gene family contained 5`UTR, 11 genes contained 3`UTR, and 8 genes did not have UTR sequences, namely *EsMBD1*, *EsMBD6*, *EsMBD7*, *EsMBD9*, *EsMBD10*, *EsMBD13*, *EsMBD15*, and *EsMBD20*. The *EsMBD* genes in the same branch of the evolutionary tree also presented similar structures. Ten conserved motifs of *EsMBD* were identified using the online tool MEME analysis (Fig. 2D) and named as motifs 1–10. Furthermore, *EsMBD17* contained only 2 motifs, the fewest in the *EsMBD* gene family, whereas *EsMBD10* contained the most 10 motifs. Over 50% of the *EsMBD* gene family contained motifs 1, 2, 6, 7 and 9, indicating that these five genes are relatively conserved in this family.

### Analysis of cis-acting elements of the *EsMBD* gene promoter

Sequence analysis of the promoter region of the *EsMBD* gene family in *E. senticosus* (Fig. 2B) revealed that four phytohormone response elements were identified in addition to the conventionally transcriptional regulatory elements of TATA-box and CAAT-box, including the methyl jasmonate (MeJA), gibberellin, salicylic acid, abscisic acid. Some environment-related transcriptional regulatory elements were also included, such as light response, anaerobic induction, drought inducibility, and low temperature. The number of light-response-related cis-acting elements was the highest in the promoter regions of all *EsMBD* genes (Supplementary Table S1). Additionally, cis-acting elements related to the regulation of flavonoid biosynthesis were found in the promoter region of *EsMBD4.* Therefore, we hypothesized that *EsMBD* might be involved in mediating the physiological regulation of *E. senticosus* and the synthesis of flavonoid substances by binding to methylated cytosine under different light conditions.

### Expression analysis and quantitative real-time PCR validation of *EsMBD* gene family

**Fig. 3:**
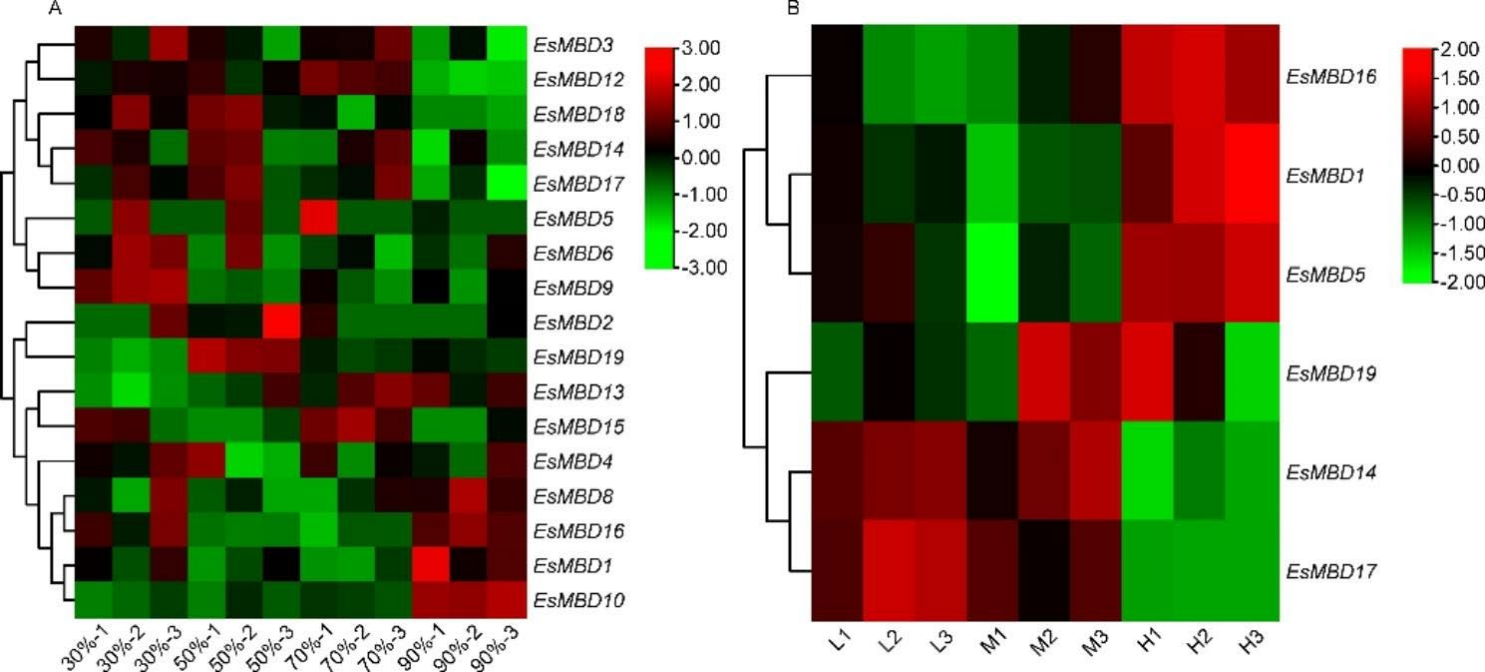
*EsMBD* gene expression in different *E. senticosus* transcriptomes. A: *EsMBD* gene expression under drought stress. B: *EsMBD* gene expression in different saponin content groups. L, low saponin content; M, middle saponin content; H, high saponin content. Three replicates were performed for each group of samples

The expression of *EsMBD* genes under drought stress in *E. senticosus* is shown in Fig. 3A. The expression of different *EsMBD* genes varied with different water contents: *EsMBD3*, *EsMBD12*, *EsMBD14*, *EsMBD17*, and *EsMBD18* showed low expression at 90% relative water content, whereas *EsMBD6* and *EsMBD9* were highly expressed at 30% relative water content, and *EsMBD1*, *EsMBD8*, *EsMBD10*, and *EsMBD16* were highly expressed at 90% relative water content. The expression of *EsMBD13* gradually decreased with increasing water content. The expression of *EsMBD* genes in the different saponin content groups is shown in Fig. 3B. *EsMBD14* and *EsMBD17* were significantly expressed in the low and medium saponin content groups but were not significantly expressed in the high saponin content group, whereas *EsMBD1*, *EsMBD5*, and *EsMBD16* were not significantly expressed in the low and medium saponin content groups but were significantly expressed in the high saponin content group.

**Fig. 4:**
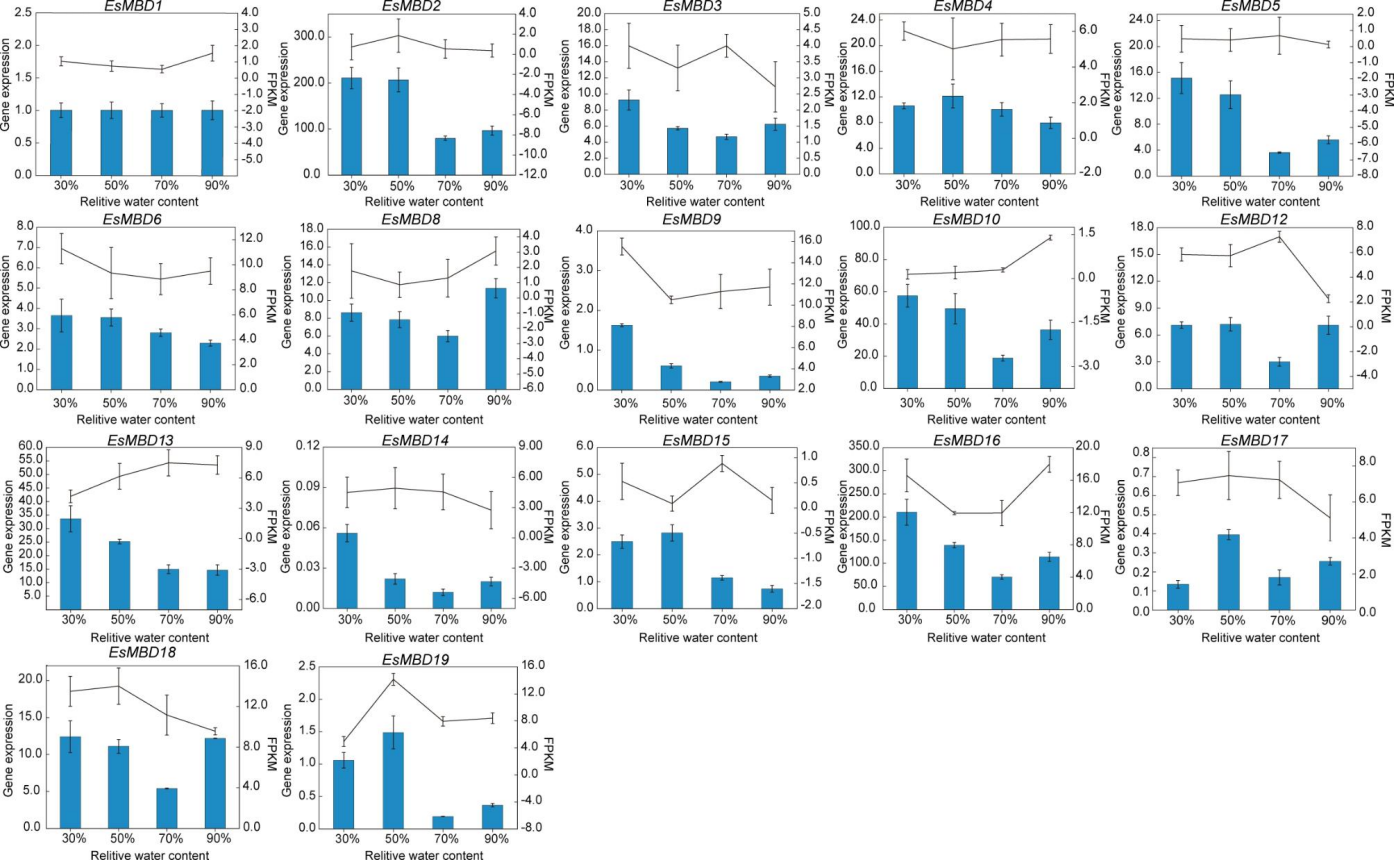
Quantitative Real-time PCR validation of *EsMBD* genes in *E. senticosus*. FPKM: Fragments Per Kilobase of exon model per Million mapped fragments

To verify the reliability of the transcriptome data, all differentially expressed genes were screened for qRT-PCR validation, except for four *EsMBDs*, which were not expressed in the transcript. The results showed that the trends of other genes were consistent with the transcriptome sequencing data, indicating that the transcriptome sequencing results were reliable (Fig. 4).

### Molecular docking of *Es*MBD proteins to DNA sequences

**Fig. 5:**
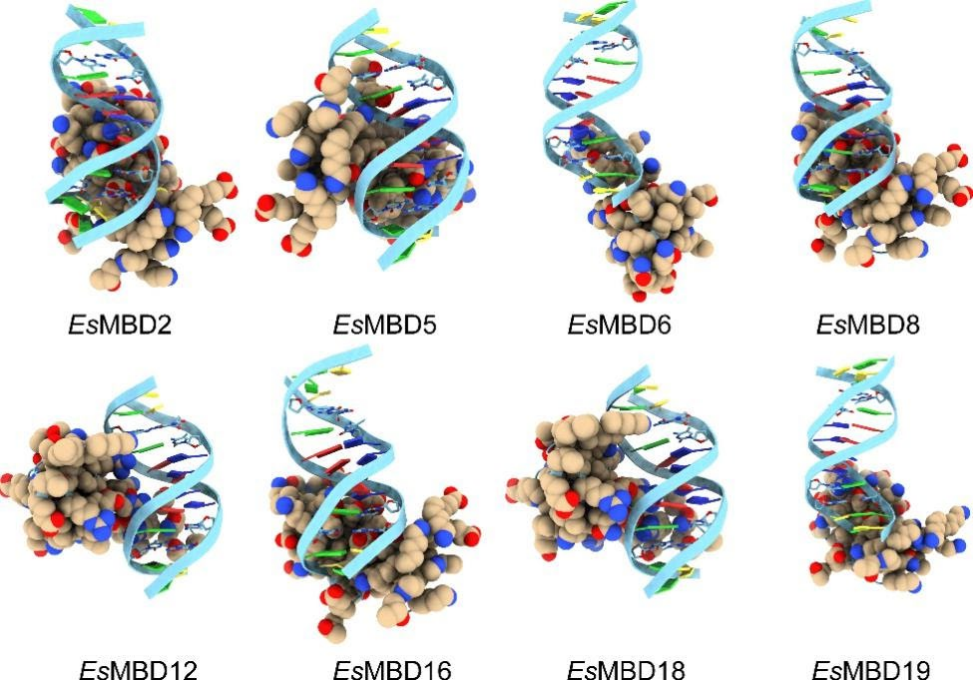
Model of *Es*MBD protein docking with methylated DNA sequences. MBDs are depicted in yellow, methylated DNA sequences are displayed in blue, and 5-methylcytosine is located between the third and ninth positions

Homology modeling of *Es*MBD proteins was performed using the SWISS-MODEL website. The GMQE ranged from 0.02 to 0.21, QMEANDisCo Global values ranged from 0.41 ± 0.11 to 0.72 ± 0.11, and sequence similarity ranged between 22.22 and 73.24%. Molecular docking was performed at the HDOCK SERVER website (Fig. 5). Among the eight best predictions acquired, each *Es*MBD protein was successfully docked to the methylated DNA sequence, indicating that *Es*MBD could bind to fully methylated CpG dinucleotides and thus play a regulatory role.

### Subcellular localization of *Es*MBD proteins

**Fig. 6:**
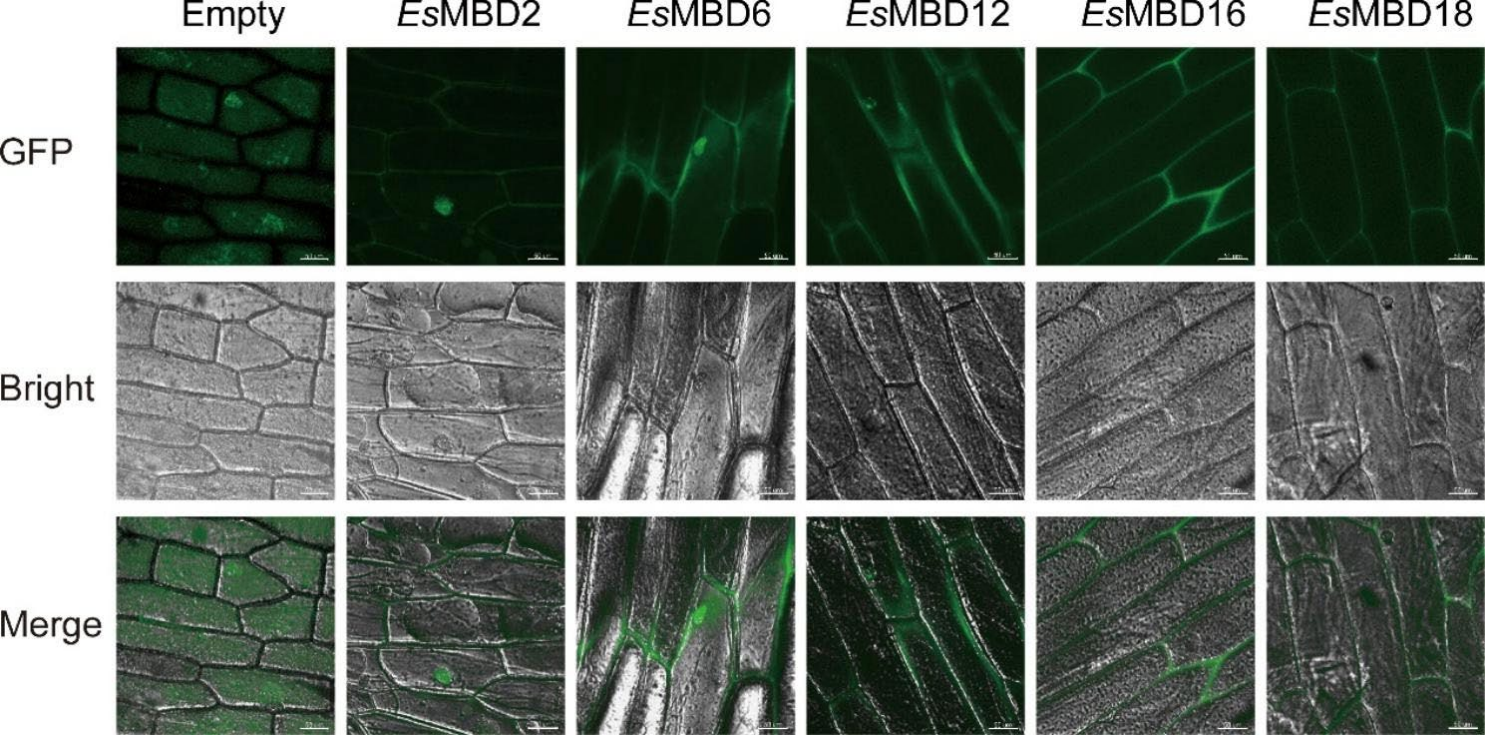
Subcellular localization of the five *Es*MBD proteins. Green fluorescence (GFP) signals were observed under a laser scanning confocal microscope to indicate the subcellular localization of *Es*MBD-GFP fusion proteins. The ruler in the image is 50 μm

Subcellular localization can reveal the working positions of proteins at the cellular level. The WoLF PSORT website (https://wolfpsort.hgc.jp) was used to predict the subcellular localization of *Es*MBD proteins. The results showed that most *Es*MBD proteins were predicted to be located in the nucleus, *Es*MBD12, *Es*MBD16, *Es*MBD17, *Es*MBD18, and *Es*MBD20 were predicted to be located in the cytoplasm, and *Es*MBD2 and EsMBD5 were predicted to be located in the nucleus and cytoplasm (Supplementary Table S2). To verify the prediction of subcellular localization, we selected 6 *Es*MBD proteins involved in molecular docking to fuse Green Fluorescent Protein (GFP) and expressed them instantaneously in *Allium cepa* L. Five *Es*MBD proteins were localized in the nucleus and cytoplasm, among which *Es*MBD2 and *Es*MBD6 were expressed in both the nucleus and cytoplasm and *Es*MBD12, *Es*MBD16, and *Es*MBD18 were expressed only in the cytoplasm (Fig. 6).

## Discussion

In the model plant *A. thaliana*, mutations are not completely random throughout the genome because of epigenetic modifications and other effects, and the mutation probability of important genes is lower than that of non-important genes [19]. *MBD* genes are inseparable from DNA methylation, and have been identified and validated in various plants. In these plants, MBDs are involved in a several physiological processes and stress regulation [20]. *MBD* genes are not only important for plant growth and development but also pose great relevance to humans [21]. In this study, we conducted a comprehensive bioinformatics analysis of *EsMBD* genes in *E. senticosus*, identified *EsMBD* family members by sequence similarity, and analyzed their evolutionary amplification patterns, sequence characteristics, expression motifs, and homology to better model them, which not only provided a basis for further studies of *MBD* genes, but also laid a foundation for epigenetic modifications affecting the genome and physiological activities of the organism.

### Evolution of *MBD* genes

Gene duplication drives the evolution of genetic systems by providing a rich source of raw material. Polyploidization is a major mechanism of environmental adaptation and species formation in organisms [22], and the major mechanism of biological genome structure and gene evolution is through a single gene, chromosome, and whole-genome duplication (WGD) [23]. The sequencing of the genome of *E. senticosus* showed that in addition to the γ genome-wide triploidization event common to all core members of the family Pentacanthaceae, *E. senticosus* also experienced two WGD events [24]; thus, it could be hypothesized that the *E. senticosus MBD* gene family expanded during these two WGD events. Further analysis of the collinearity between *E. senticosus* and chromosomal localization information revealed that the genomic collinearity level of *E. senticosus* was as high as 62.05%, and there was also a significant collinearity among *EsMBD* gene families, while the *EsMBD* gene families were scattered in arrangement on chromosomes, and only one tandem repeat gene existed. Therefore, it could be inferred that the main method of amplification of *EsMBD* gene families was gene fragment duplication. The collinearity analysis showed significant collinearity between *E. senticosus* and *A. elata*, which are both species of Araliaceae, while there was also significant collinearity between the *EsMBD* and *AeMBD* genes. Therefore, it can also be inferred that *MBD* genes evolved within the Araliaceae species before eventually turning into interspecies-specific *MBD* genes through amplification and duplication.

### Phylogeny of the *MBD* gene family

MBD proteins are key determinants of DNA methylation status [25]. Twenty *EsMBD* genes and ten *AeMBD* genes were identified in *E. senticosus* and *A. elata*. Together with the 13 *AtMBD* genes in *A. thaliana*, a phylogenetic tree was constructed and branched according to a previously constructed phylogenetic tree between *Brassica napus* L. and *A. thaliana* [26]. Although there was only *AtMBD8*, *EsMBD4*, and *EsMBD20* in one branch, the *MBD* gene family members of *E. senticosus* and *A. elata* were identified in all the other branches. This might be attributed to the fact that the relatives of *E. senticosus* being closer to each other than those of *A. thaliana* and *A. elata*. The phylogenetic tree was also verified using the inter-species collinearity map of *E. senticosus* and *A. elata*. *MBD* gene family members have different abilities to bind to methylated DNA [27]. Therefore, we speculated that the functional differences in *MBD* genes among different branches might be significant, indicating that the functional differences among MBD proteins were gradually realized during the evolutionary process. This result is consistent with the conclusions of previous studies that *MBD* genes have a long evolutionary history, with *MBD* genes existing and beginning to differentiate as early as the formative stages of monocotyledons and dicots [26].

### Structure of *MBD* genes and protein-conserved motifs

UTRs are closely associated with gene expression motifs that enhance plant perception of stimuli from developmental and environmental regulatory factors [28]. In the *MBD* gene family of *E. senticosus*, UTRs were present in 12 genes, accounting for 60% of all *EsMBDs. EsMBDs* lacking UTR structures were in the same branch or adjacent ones, implying that the expression motifs of the *MBD* gene family shared some similarity between the same and adjacent branches, whereas the structures and differences of genes were more significant between different branches. Studies have shown that dehydration stress can induce the extension of the 3’UTR transcript, and that these stress-induced 3’UTR extensions have a new function: regulating the long non-coding RNA of its adjacent genes, which confirms the importance of UTR length in plant adaptation to stress conditions [29]. The UTR lengths of different *MBD* genes in *E. senticosus* were different, which also indicated that different genes may have functional differences and play different roles under various stresses.

*EsMBD* sequences exhibit significant conservation and inter-group specificity across adjacent branches, and conserved structural sequences are prerequisites for conserved biological functions [30]. The *EsMBD* motifs were similar in structure between adjacent branches but differed significantly between branches that were further apart from each other. It was speculated that this was due to the different functional differentiation of *MBD* genes during the process of evolution. Moreover, the 20 *EsMBD* protein sequences obtained from the screening also presented conserved motifs in most *EsMBDs*, which is consistent with the results of earlier studies comparing conserved motifs of *MBD* in *B. napus* [26]. These conserved motifs of different *MBD* genes in *E. senticosus* also showed different similarities.

### Promoter analysis of the *MBD* gene family

A promoter is a small region of a DNA sequence that responds to various transcription factors and triggers specific gene expression [31]. Cellular metabolism in organisms is largely regulated at the transcriptional level, and promoters are responsible for basic regulation of transcription initiation [32]. The promoters of the *MBD* genes in *E. senticosus* were found to contain a large number of cis-acting elements, and the quantity of different cis-acting elements in adjacent branches showed some similarity, suggesting that there is a certain degree of functional differentiation among the *MBD* genes in *E. senticosus*, which is consistent with a previous hypothesis that there is functional differentiation of *MBD* genes during the evolutionary process [26]. Among the various cis-acting elements of the promoter, light response elements were the most abundant, followed by anaerobic induction elements, indicating that the *EsMBD* genes of *E. senticosus* were subjected to maximum regulation by light and anaerobic conditions (Supplementary Table S1). It has been demonstrated that different light qualities like light intensities regulated the growths of roots, stems, and leaves as well as the synthesis of secondary metabolites in *E. senticosus* [33, 34]. For example, red light was found to induce the synthesis of Eleutheroside E [33] and LED lighting promoted the development of fine roots in *E. senticosus* seedlings [34]. In addition, shade treatment enhances photosynthesis in *E. senticosus* by promoting the growth and accumulation of secondary metabolites [35]. The *EsMBD* promoter regions, which contain substantial light-responsive and cis-acting elements involved in the regulation of flavonoid synthesis, were also identified. We speculated that the *EsMBD* gene of *E. senticosus* could participate in regulating the physiological activities of *E. senticosus* under certain light stress conditions, thereby affecting the primary and secondary metabolism of *E. senticosus*.

### *EsMBD* gene expression analysis

Drought stress alters plant growth and developmental processes, causing morphological changes in plant height and leaf size. Plants also respond to drought stress by reducing water loss and enhancing water absorption capacity [36]. It has been suggested that drought induced increases in DNA methylation may be one of the main mechanisms by which plants respond to drought stress [37]. In maize, the *MBD* genes of *Zea mays* L. showed a similar expression motif under drought stress [38]. Interestingly, *EsMBD* genes in *E. senticosus* also exhibited similar expression motifs under drought stress, with some *EsMBD* gene expression showing similar up- or down-regulation trends at different relative water contents, and most *EsMBD* genes were up-regulated under relatively dry conditions. In a previous study by Cui et al. [7], the saponin content of *E. senticosus* decreased with increasing DNA methylation ratio. Meanwhile, *EsMBD14* and *EsMBD17* were significantly expressed in the low and medium saponin content groups but were not significantly expressed in the high saponin content group. This indicates that *EsMBD14* and *EsMBD17* were not significantly expressed in the low methylation ratio group. This suggests that *EsMBD14* and *EsMBD17* are involved in regulating saponin synthesis under drought stress through DNA methylation.

### Molecular docking model analysis and subcellular localization of *EsMBD* genes

The MBD protein of *E. senticosus* was revealed using homology modeling on the SWISS-MODEL website. The Global Model Quality Estimate [39] (GMQE) is a quality assessment criterion that integrates the properties of the target template alignment and template structure to improve the reliability of the quality estimate. The QmeandisCo global score [40] is the average QMEANDisCo score for each residue. Based on the QMEANDisCo score estimated for substantial models, it provides an error estimate and represents the standard deviation between the QMEANDisCo global score and underlying facts. Because the reliability of the predictions depends on the model size, the provided error estimates are calculated in line with models of sizes similar to the inputs. The global model quality estimates, together with the QMEANDisCo global score demonstrated both the accuracy and reliability of the prediction models. In homology modeling, *Es*MBD protein models were derived from the MBD proteins of *Homo sapiens*, and *A. thaliana* showed the highest sequence similarity of 73.24%. This confirmed our previous hypothesis that *MBD* genes evolve and are amplified within the *E. senticosus* genome. Among all *Es*MBD protein homology models, the size and morphology varied constantly. Combined with the results of motif analysis, it can be speculated that there are different degrees of functional differentiation among *Es*MBDs, whereas the study in *Glycine max* (Linn.) Merr. [41] verified that most MBDs were differentiated during early evolution, which was also consistent. MBD proteins can specifically bind to methylated cytosines, thus causing DNA methylation silencing in methylated genes [9]. All those screened from *E. senticosus* were able to dock successfully with methylated DNA after homology modeling, which not only verified the basic mode of action of MBD proteins but also predicted *Es*MBD binding to methylated functional genes as one of the pathways of DNA methylation regulating secondary metabolism in *E. senticosus*. Among all models, *Es*MBD2 and *Es*MBD5 presented the best quality and highest sequence similarity. To further verify the function of *Es*MBD proteins, we examined the subcellular localization of 5 *EsMBDs* that responded strongly to drought stress. Previous studies have shown that AtMBD5 protein is located in the nucleus and interacts with AtRAN3 protein [42]. In our study, all *Es*MBDs were located in the nucleus and cytoplasm, consistent with the results of previous studies. *Es*MBD proteins are widely involved in the physiological metabolism of *E. senticosus* and play important roles in its stress response and secondary metabolic processes. This also showed that *E. senticosus* is likely to affect the synthesis of saponins and other substances through epigenetic modifications under drought stress.

## Conclusion

In this study, 20 *EsMBD* genes were identified in *E. senticosus*, which were mainly obtained by fragment duplication and found to be unevenly distributed on 12 of the 24 chromosomes. The gene structure and conserved motifs of *EsMBD* genes exhibited different degrees of similarity, with the greatest degree between adjacent branches of the evolutionary tree and substantial light-, hypoxia-, and drought-related cis-acting elements in the *EsMBD* promoter. Under drought stress, *EsMBD* genes of *E. senticosus* showed similar expression motifs. Comparing the transcriptome data and Quantitative Real time PCR results of different saponin content groups of *E. senticosus*, it was found that different *EsMBD* genes had different sensitivity to drought stress, among which *EsMBD14* and *EsMBD17* were most sensitive to drought stress. After molecular docking model analysis and subcellular localization, it can be inferred that functional differentiation occurred during the evolutionary process of *EsMBDs*. Based on the above analysis and transcriptome data, the cytosine of the functional gene promoter in the *E. senticosus* genome can be effectively methylated under various stress conditions. *EsMBD14* and *EsMBD17* combine with methylated cytosine, resulting in changes in the expression of functional factors, thus affecting the secondary metabolism process of *E. senticosus*.

## Materials and methods

### Identification and physicochemical characterization of the *EsMBD* gene family

Whole genome sequencing data for *E. senticosus* [24] were downloaded from the CNGB Sequence Archive (CNSA) database (https://ftp.cngb.org/pub/CNSA/data3/CNP0001682/CNS0348054/CNA0019321/). Whole genome sequencing data of *A. elata*e [43], which is the same species of Araliaceae, were downloaded from the Dryad database (https://datadryad.org/stash/dataset/doi:10.5061/dryad.69p8cz937). The Hidden Markov Model (PF01429) for *MBD* gene was downloaded from the PFAM database (http://pfam.xfam.org/). The structural domains of the MBD proteins were searched for using the HMMER program, and the E value was set to 0.001. Furthermore, the online Simple Modular Architecture Research Tool (SMART) and NCBI Batch CD-Search tool were used to confirm the conserved domains of all the candidate MBD protein sequences. We retained only sequences containing significant MBD domains in all results; therefore, the sequence of *E. senticosus* was eliminated. *MBD* gene family members were identified from the whole genomes of *E. senticosus* and *A. elata*, and named according to their different positions on the chromosome (Supplementary Table S3). The obtained *Es*MBD protein sequences were submitted to ProtParam (https://web.expasy.org/protparam/) and analyzed for physicochemical properties, such as amino acid content, molecular weight, isoelectric point, hydrophilicity, and instability coefficient.

### Chromosomal localization and collinearity analysis of *EsMBD* genes

For collinearity analysis, *E. senticosus* genome files and *E. senticosus* genome files with *A. elata* were used for sequence alignment using the Basic Local Alignment Search Tool (BLAST), with a cut-off E value of 1.0 e^− 10^. The MCScanX software was used to perform collinearity analysis based on BLASTP. TBtools [44] (https://github.com/CJ-Chen/TBtools) software was used to visualize collinearity.

### Phylogenetic analysis of *MBD* genes

The 13 *At*MBD protein sequences of *A. thaliana* were downloaded from The Arabidopsis Information Resource (https://www.arabidopsis.org/). Twenty *Es*MBD proteins identified in *E. senticosus*, 12 *Ae*MBD proteins from *A. elata*, and 13 *At*MBD proteins from *A. thaliana* were used to construct the phylogenetic tree. We used the MBD gene family of *A. thaliana* as an outgroup, according to previous methods [26], and constructed a phylogenetic tree with *A. elata* to obtain more accurate results. ClustalW was used for multiple sequence alignment of MBD protein sequences with default parameters. The alignment results were used to construct an NJ phylogenetic tree using MEGA 11.0. Bootstrap resampling (100) was used to assess the reliability of interior branches. The bootstrap value was set at 1,000. The online tool EvolView (https://www.evolgenius.info/evolview/) was used to construct a phylogenetic tree.

### Gene structure and sequence analysis of the *EsMBDs*

The exon-intron structure analysis was performed using TBtools software by inputting gene annotation GFF files. The online MEME Suite was used to confirm the motifs of *E. senticosus* EsMBD protein sequences with the following parameters: maximum number of ten motifs and optimum width of 6–50.

### *EsMBD* gene promoter’s cis-acting element analysis

The 2,000 bp nucleotide fragment upstream of the start codon of each *EsMBD* gene in the *E. senticosus* genome was screened as the promoter sequence. The cis-acting element analysis of these *EsMBD* genes was performed using the online software PlantCARE (http://bioinformatics.psb.ugent.be/webtools/plantcare/html/).

### Expression analysis of *EsMBD*

The transcriptome sequencing data of drought-stress-treated *E. senticosus* were downloaded from the NCBI database (Accession: SRR19962743-SRR19962757). Based on the results of a previous study by Cui et al. [7], transcriptome data of *E. senticosus* with different saponin contents (Accession: SRX13417593-SRX13417601) were downloaded from the NCBI database. Based on the different transcriptome data, differentially expressed *EsMBD* genes were screened (Supplementary Table S4-S5). The expression values (FPKM values) were used to compare the transcript levels of MBD in *E. senticosus*. The transcripts with log2FC ≥ 1.5 or ≤ -1.5 and FDR < 0.05 were selected as transcripts that were differentially expressed. Heatmaps of *EsMBD* gene expression were drawn using the Heatmapper website (http://www.heatmapper.ca/), based on the expression of differentially expressed genes.

To verify the accuracy of the expression levels obtained from RNA-Seq analysis, the screened *EsMBD* was validated by qRT-PCR. Primers for qRT-PCR amplification of differentially expressed *EsMBD* genes were designed using the Prime Premier software (5.0) (Supplementary Table S6). The specificity of the qRT-PCR primers was determined by analyzing the solubility curves. The qRT-PCR was performed using Talent qPCR PreMix (SYBR Green) (TIANGEN, Beijing) on an Applied Biosystems 7900HT PCR System (THERMO FISHER SCIENTIFIC, Waltham, MA, USA) with the *glyceraldehyde-3-phosphate dehydrogenase* gene (*GAPDH*) used as an internal reference gene [45]. Three biological replicates were used for each sample. The total reaction system was 10.0 µL with 1.0 µL enzyme, 2.9 µL Nuclease-Free Water, 0.3 µL each primer, 0.5 µL cDNA, 1.0 µL ROX Refernence Dye. The reaction conditions were as follows: pre-denaturation at 95 °C for 3 min, denaturation at 95 °C for 5 s, annealing at 55 °C for 10 s, and supplemental extension at 72 °C for 15 s. The reaction was completed after 40 cycles. *EsMBD* gene expression was calculated using SDS 2.4 software through the 2^−ΔΔ^ Ct method [46].

### Molecular docking of *Es*MBD proteins to DNA sequences

Homology modeling of *Es*MBD proteins was performed using SWISS-MODEL (https://swissmodel.expasy.org/) with the Global Model Quality Estimate [39] (GMQE), QMEANDisCo [40] global score, and sequence similarity ratio. The methylated DNA sequence models (PDB ID: 5UZ2) were downloaded from the RCSB PDB website (https://www.rcsb.org/). *Es*MBD proteins and methylated DNA sequences were docked on the HDOCK SERVER website (http://hdock.phys.hust.edu.cn/). The molecular docking results were obtained using the UCSF ChimeraX website (https://www.cgl.ucsf.edu/chimerax/).

### Vector construction

The cloning and restriction primers were designed using Prime premier 5.0 (Supplementary Table S7) [39]. The *EsMBD* sequences were cloned using cloning primers and enzyme digestion primers, using *E. senticosus* cDNA as a template and *Taq* DNA polymerase (TIANGEN, Beijing). The amplification conditions were as follows: pre-denaturation at 94 °C for 5 min, denaturation at 94 °C for 30s, annealing at 55 °C for 30s, extension at 72 °C for 5 min, and an additional extension at 72 °C for 5 min. A lethal fast cloning kit (TIANGEN, Beijing) was used to connect the cloned sequences with the pLB vectors and was introduced into competent *Escherichia coli* Top10 cells (TIANGEN, Beijing, China). Sequencing was performed using a SinoGenoMax (Beijing, China). The recombinant pLB plasmids were extracted using a TIANprep Mini Plasmid Kit (TIANGEN, Beijing, China). The pHG vectors and recombinant pLB plasmids were digested overnight at 37 °C with BamHI-HF, PstI-HF, and SacI-HF (NEW ENGLAND BIOLABS, Beijing, China) restriction enzymes. We used T4 DNA ligase (NEW ENGLAND BIOLABS, Beijing, China) to connect *EsMBD* genes with pHG vectors overnight at 16 °C to construct recombinant *EsMBD-GFP* expression vectors. The total reaction system was 20.0 µL with 1.0 µL T4 DNA Ligase, 1.0 µL Nuclease-Free Water, 2.0 µL T4 DNA Ligase Buffer, 11.0 µL Insert DNA (20 ng / µL), 5.0 µL pHG vector.

### Subcellular localization of *Es*MBD proteins

We selected *Allium cepa* L. as the infection receptor. The recombinant expression vectors were transformed into *Agrobacterium tumefaciens* (Biomed, Beijing, China) and cultured on LB solid medium containing kanamycin (100 ng/ml) and rifampicin (50 ng/ml). The positive colonies were selected and cultured overnight in LB liquid medium at 28 °C. The cells were then centrifuged at 3000 rpm for 10 min to collect the cells, which were resuspended in LB liquid medium without antibiotics. The medium contained MgCl_2_ (10 mmol/L) and acetosyringone (100 mmol/L), and was adjusted to an OD_600_ of 1.0.

To prepare LB solid culture medium, the outer skin of the onion was peeled off without antibiotics. Fresh and thick scales were taken and a small square with an area of 1 cm^2^ on the inner skin was cut using scissors. The onion skin was torn off with tweezers, placed downward close to the mesophyll in LB solid culture medium without antibiotics, and sealed with a sealing film for dark culture at 28 °C for 24 h. After 24 h, the inner skin of *A. cepa* was removed and soaked in LB heavy suspension solution at room temperature for 20 min. After 20 min, tweezers were used to collect the epidermis, filter out the bacterial liquid, lay on new LB solid medium without antibiotics, and incubated for 16–24 h at 25 °C with a photoperiod of 16 h/8 h. Small pieces of onion inner skin were removed, shaken, washed with clean liquid LB medium to remove attached *A. tumefaciens*, placed on a slide, and observed under a laser scanning confocal microscope (Leica, Germany).

## Electronic supplementary material

Below is the link to the electronic supplementary material.

**Additional file 1. Table S1.** *EsMBDs* promoter region cis-acting element. **Table S2.** WoLF PSORT Predicts Subcellular Localization of *EsMBD* Proteins. **Table S3.** Methyl-binding domain in the genome of *Eleutherococcus senticosus* (Rupr. et Maxim) Maxim and *Aralia elata* (Miq.) Seem. **Table S4.** Expressed MBD genes in *Eleutherococcus senticosus* (Rupr. et Maxim) Maxim of different relative water content. **Table S5.** Expressed MBD genes in *Eleutherococcus senticosus* (Rupr. et Maxim) Maxim of different saponin content. **Table S6.** Primers for qRT-PCR. **Table S7.** Primers for Vector construction and Subcellular localization.

## Data Availability

The data that support the findings of this study are available from the NCBI database (Accession: SRR19962743-SRR19962757) but restrictions apply to the availability of these data, which were used under license for the current study, and so are not publicly available. Data are however available from the authors upon reasonable request and with the permission of Zhaobin Xing (xingzb@ncst.edu.cn).
